# A Terahertz (THz) Single-Polarization-Single-Mode (SPSM) Photonic Crystal Fiber (PCF)

**DOI:** 10.3390/ma12152442

**Published:** 2019-07-31

**Authors:** Tianyu Yang, Can Ding, Richard W. Ziolkowski, Y. Jay Guo

**Affiliations:** Global Big Data Technologies Centre (GBDTC), University of Technology Sydney (UTS), Ultimo, NSW 2007, Australia

**Keywords:** epsilon-near-zero (ENZ), fiber characterization, photonic crystal fiber (PCF), single-polarization-single-mode (SPSM), terahertz (THz)

## Abstract

This paper presents a novel approach to attain a single-polarization-single-mode (SPSM) photonic crystal fiber (PCF) in the terahertz (THz) regime. An initial circular hole PCF design is modified by introducing asymmetry in the first ring of six air holes in the cladding, i.e., epsilon-near-zero (ENZ) material is introduced into only four of those air holes and the other two remain air-filled but have different diameters. The resulting fundamental X-polarized (XP) and Y-polarized (YP) modes have distinctly different electric field distributions. The asymmetry is arranged so that the YP mode has a much larger amount of the field distributed in the ENZ material than the XP mode. Since the ENZ material is very lossy, the YP mode suffers a much higher loss than the XP mode. Consequently, after a short propagation distance, the loss difference (LD) between the XP and YP modes will be large enough that only the XP mode still realistically exists in the PCF. To further enhance the outcome, gain material is introduced into the core area to increase the LDs between the wanted XP mode and any unwanted higher order (HO) modes, as well as to compensate for the XP mode loss without affecting the LD between the XP and YP modes. The optimized PCF exhibits LDs between the desired XP mode and all other modes greater than 8.0 dB/cm across a wide frequency range of 0.312 THz. Consequently, the reported PCF only needs a length of 2.5 cm to attain an SPSM result, with the unwanted modes being more than 20 dB smaller than the wanted mode over the entire operational band.

## 1. Introduction

Polarization-maintaining photonic crystal fibers (PM PCFs) have been widely used in various applications, such as precision optical instruments, optical communication systems, and sensors [1]. There are basically two main types of PM PCFs, i.e., highly birefringent (HB) PCFs and single-polarization-single-mode (SPSM) PCFs. To date, a variety of HB PCFs with birefringence values ranging from 0.001 to 0.1 have been proposed and studied both theoretically and experimentally [2,3,4,5,6,7]. On the other hand, there have been many fewer studies on SPSM PCFs, especially in the terahertz (THz) frequency regime. 

There are generally two approaches to achieving a PCF with an SPSM outcome. One is to use index-matching coupling techniques. By introducing index-matched cladding defects, one can arrange the attenuation rates of two orthogonal modes to be very different and, thus, suppress the mode with the higher attenuation rate after some propagation distance [8]. The second approach is to introduce physical asymmetries into the PCF configuration, e.g., to use ellipse-shaped air holes to make the cut-off frequencies of the resulting two fundamental polarization modes be different. In this manner, only the higher index mode for the range between those two cut-off frequencies will be supported in the fiber core, again after some propagation distance [9]. 

Despite the recent significant rise in interest in THz communications, most of the reported SPSM PCFs to date have been developed for optical applications [8,9,10,11,12]. Only a few SPSM PCF designs have been developed for the THz regime [13,14]. Low technology readiness levels (TRL) of appropriate materials and sources have slowed progress at these frequencies. Nevertheless, SPSM PCFs have wide applicability in a variety of THz guided wave applications. 

As noted, the key point in designing an SPSM PCF is to achieve a minimum loss difference (MLD) between the wanted and all other (unwanted) modes. The total loss (TL) in a PCF is generally taken to be the sum of the confinement loss (CL) and the effective material absorption loss (EML). Available optical SPSM PCFs usually have similar EMLs for the two fundamental propagating modes. Hence, SPSM operation is realized by introducing differences in their CLs [8,9]. On the other hand, the TL is usually dominated by the EML in the THz regime. This fact makes it difficult to produce a large enough MLD between the two propagating polarization modes with conventional methods that focus on manipulating the CLs. For example, a THz SPSM PCF based on index-matching techniques was reported in [13]. This PCF achieves an SPSM operation in a wide frequency range (0.13 THz) from 1.67 to 1.8 THz. However, the MLD is small, only 0.036 dB/cm. By asymmetrically introducing a rectangular array of micro-circular air holes in the core region, SPSM operation with an ultra-wide bandwidth of 0.32 THz from 0.82 to 1.14 THz was attained in [14], but with only a slightly larger MLD, 0.06 dB/cm. 

In this paper, a different approach to realize SPSM propagation in a THz PCF is developed. This approach is based on a unique design that realizes different EMLs for the two fundamental propagating modes and ensures large propagation losses for the higher order (HO) modes. Previous studies have shown that different epsilon-near-zero (ENZ) materials [15] or the liquid toluene [16] can be inserted into selected air holes of a PCF to manipulate the local field distributions in it and to introduce asymmetries that facilitate eliminating the degeneracy of its two fundamental modes to achieve high birefringence properties. However, these studies also warned of the fact that using ENZ materials, which are themselves usually very lossy [17], could yield PCFs with nontrivial propagation losses. This drawback is taken as an advantage in attaining SPSM propagation in this work. The circular air hole PCF considered in [15] is the base design. By depositing ENZ material rings into a few of the first set of air holes surrounding its core and adjusting the diameter of the remaining air filled ones, the ratio of the fields associated with the two fundamental polarization modes distributed in and away from the ENZ-augmented holes is engineered by adjusting the configuration. The result is significantly different EMLs for those two modes in the optimized asymmetric PCF structure. Moreover, it is demonstrated that the MLD between the wanted mode and any unwanted HO modes is increased by doping the core area with gain material [18]. The addition of the gain material also helps to compensate for the losses introduced in the wanted mode in the presence of the lossy ENZ material. Parameter studies of the key design parameters are presented to illustrate how the configuration can be optimized to have the best performance. The simulation results demonstrate that the optimized PCF enables SPSM propagation across a wide bandwidth of 0.312 THz from 0.996 to 1.308 THz, over which the MLD between the wanted and all the other unwanted modes is above 8.0 dB/cm. Consequently, the reported PCF only needs a length of 2.5 cm to attain an SPSM result, with the unwanted modes being more than 20 dB smaller than the wanted mode over the entire operational band.

## 2. THz PCF Configuration

**Figure 1:**
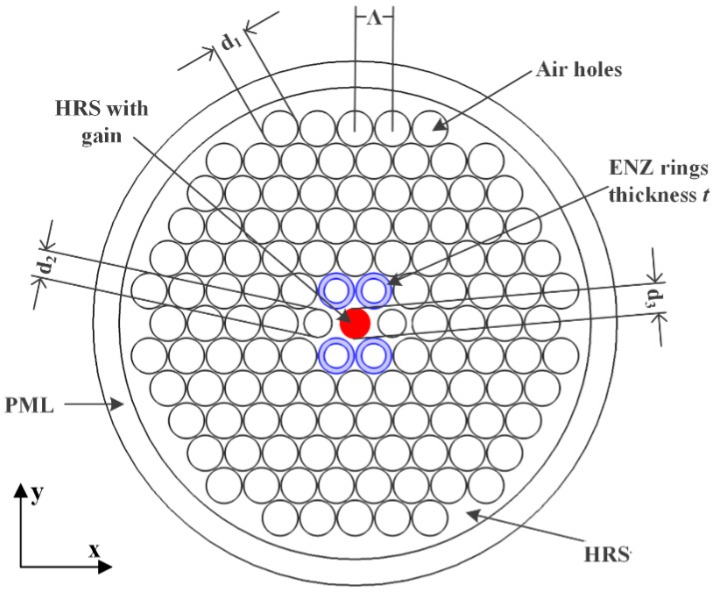
Cross sectional view of the proposed terahertz (THz) single-polarization-single-mode (SPSM) photonic crystal fiber (PCF). ENZ, epsilon-near-zero; PML, perfectly matched layer; HRS, high resistivity silicon.

Figure 1 shows a cross-sectional view of the proposed THz SPSM PCF configuration. The PCF has a solid core and a traditional cladding defined by five rings of triangularly distributed, circular air hole arrays, which are easy to fabricate. As shown in the figure, only two circular holes in the first ring surrounding the core have the diameter *d*_2_. All the other holes in the cladding have the same diameter, *d*_1_. The lattice constant, Λ, represents the distance between the centers of any two adjacent holes in the cladding. A matched layer whose thickness is 10% of the whole diameter of the PCF is set concentrically on the outside of the hole-based cladding region. The ENZ material is deposited as annular rings, highlighted in blue, in the air holes indicated in Figure 1. The thickness of the ENZ rings is *t*. These rings could be inserted into these air holes using high-pressure chemical vapor deposition, and their thickness can be controlled by the deposition time [19]. The red circular area located in the center of the core with a diameter of *d*_3_ is the region to be doped with gain material.

The background material is high resistivity silicon (HRS). This material was selected because of its outstanding properties in the THz regime, e.g., a low bulk material absorption loss, <0.015 cm^−1^, from 0.1 to 1.5 THz and a stable refractive index, 3.417, from 0.5 to 4 THz [20]. Materials with ENZ properties can be attained with metamaterials [21] or naturally existing materials, e.g., highly pure KCL [17] or AZO [22]. In accordance with the data reported for these materials, the refractive index of the ENZ rings used in this work is set to be a constant: $n_{enz}=0.2-0.3\;j$ across the frequency range of interest. Note that the time convention, *exp(jωt)*, has been assumed throughout. Similarly, as reported in [23], gain materials, e.g., rare earth materials (*Er*^3+^) [18,24] or graphene [25], can be used in fibers to compensate for losses. In this work, we assume that the gain material is lightly doped into the background material in the indicated circular core region. The resulting refractive index of this gain region is $n_{g}=3.417-\xi \;j$, where 3.417 is the relative permittivity of the background HRS and *ξ* is a negative number representing the gain factor attributed to the presence of the gain material doping it. 

The simulations in this work were conducted with the COMSOL Multiphysics commercial software package 5.3 [26]. The electric (E-) field results were obtained with COMSOL’s full-wave vector finite element method (FEM) solver. The obtained modes were calculated with its Eigen-mode solver. Since the FEM solver is an iterative algorithm, the convergence value was set to 10^−6^ and was met in all of the simulations. With this convergence value specification and with the adaptive meshing provided by the software, the minimum mesh edge length was 0.12 μm, which is about 0.0004λ at 1.0 THz. Moreover, perfectly matched layer (PML) boundary conditions were adopted for the simulations to absorb any numerical reflections arising from its outer boundaries. The thickness of the PML region was set to be 10% of the whole diameter of the PCF. COMSOL’s automated adaptive meshing algorithm determined the actual number of mesh cells in it based on that thickness and the wavelengths of interest. To ensure the absence of any numerical reflections from the PML with this choice, several simulations were performed with PML thicknesses ranging from 7% to 14% of the PCF diameter. No effects on the calculated results were observed.

## 3. Working Mechanisms

### 3.1. Passive PCF

**Figure 2:**
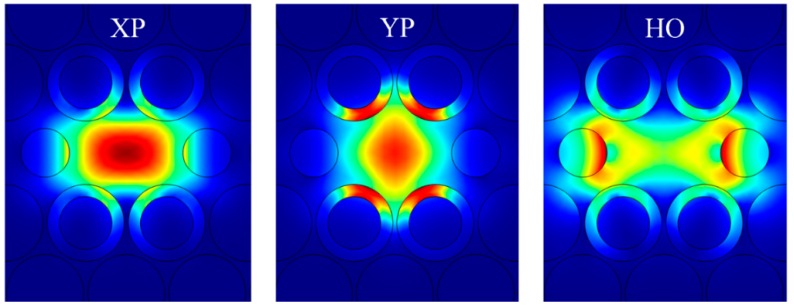
Simulated distributions of the magnitude of the total electric field of the X-polarized (XP) and Y-polarized (YP) modes and the lowest loss higher-order (HO) mode of the passive ENZ-based PCF at 1.0 THz.

To provide insights into how the proposed PCF works, Figure 2 shows the magnitude of the total E-field distribution (referring to the axes in Figure 1) of the X-polarized (XP) and Y-polarized (YP) modes and the lowest loss HO mode of an example passive PCF (without gain material in the core region). Its dimensions were set to *d*_1_ = 0.95Λ, *d*_2_ = 0.7Λ, *d*_3_ = 0.75Λ, and *t* = 0.15Λ, where Λ = 70 μm. There are several HO modes present in this PCF due to its geometrical structure. However, we have elected to only show results for the lowest loss HO mode and not to discuss the other HO modes explicitly to simplify the presentation. The HO modes have little impact on the outcome. In particular, considering the first three HO modes, the effective index of the HO 1 mode is much higher than that of the next two HO modes over the entire frequency band. Moreover, the TLs of the HO 2 and 3 modes are significantly larger than the HO 1 mode for frequencies greater than 1 THz and, hence, over most of the frequency range of interest. Furthermore, there is very little electric field distributed in the core region where the gain material resides for any of the HO modes. Consequently, the HO 1 mode dominates all other HO modes with respect to the loss difference (LD) between the XP and any other HO modes. Therefore, if the final results with respect to the HO 1 mode satisfy the LD requirements, all other HO modes also will. 

As shown, the E-fields of the three modes are distributed in both the core and in the circular holes filled and not filled with the ENZ rings. However, there are clearly different relative amounts in each region for each mode. Since the signal propagation experiences a much higher loss when its fields are in the ENZ rings than in the core or air hole regions, the resulting local field distributions lead to a different attenuation rate for each mode. For example, it is clear from Figure 2 that the YP mode has the highest portion of its E-field confined to the ENZ rings and, hence, it has the largest attenuation rate. On the other hand, the XP mode has its E-field mainly confined to the core region and thus has a smaller attenuation rate. The E-field of the lowest loss HO mode is concentrated in the core area away from its center, around the edges of the air holes.

**Figure 3:**
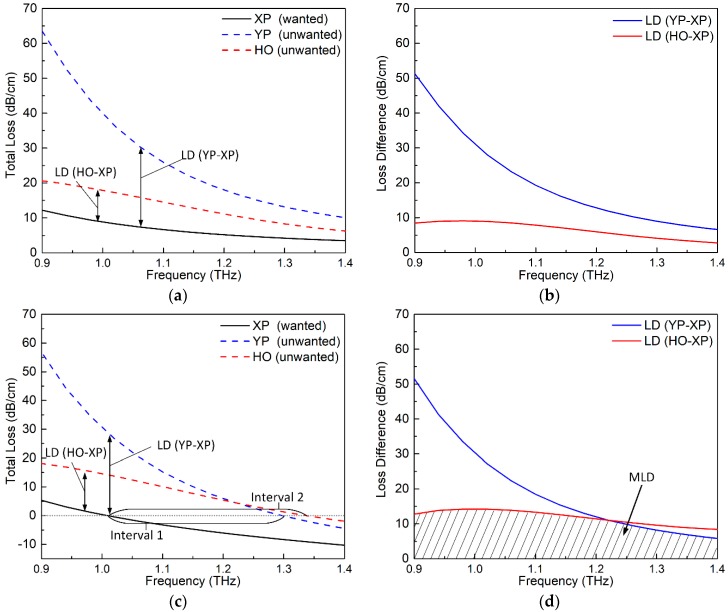
Simulated SPSM PCF results. (**a**) total losses (TLs) and (**b**) loss differences (LDs) of the passive version. (**c**) TLs and (**d**) LDs of the active version.

The loss factors of the three modes of the passive PCF illustrated in Figure 2 were calculated and are plotted in Figure 3a. The TL considered in this work is the sum of CL and EML. The CL is calculated as [27]

(1) $$L_{c}\left(cm^{-1}\right)=\frac{4\pi f}{c}\times Im\left[n_{eff}\right]$$

where *c* is the speed of light in a vacuum, and *Im*[*n_eff_*] is the imaginary part of the effective refractive index. The EML is calculated as [28]

(2) $$\alpha_{eff}\left(cm^{-1}\right)=\;{\left(\frac{\varepsilon_{0}}{\mu_{0}}\right)}^{\frac{1}{2}}\;\;\frac{\int_{mat}^{}n_{mat}\alpha_{mat}{\left|E\right|}^{2}dA}{2\int_{All}^{}S_{z}dA}$$

where ε_0_ and μ_0_ are the permittivity and permeability of vacuum, α_mat_ is the bulk material absorption loss, *n_mat_* is the refractive index of the material, *E* is the modal electric field, and *Sz* is the Poynting vector projected along the propagation (*Z*) direction. 

As shown in Figure 3a, the XP mode, which is the only desired mode for the SPSM outcome, has the smallest loss factor. The YP and HO modes, which are the unwanted modes, have larger loss factors. Among the two unwanted modes, the YP mode exhibits the largest loss factor since it has the largest portion of its electric field being confined in the ENZ rings. These results are in accordance with the previous qualitative deductions from the E-field distributions shown in Figure 2. To have a clearer view of the attenuation differences between the different modes, Figure 3b further plots the LDs between the unwanted modes and the wanted mode. The larger the LD is, the shorter the fiber needed to achieve the desired SPSM propagation. It can be seen from the figure that the LDs decrease as the frequency increases. In addition, the LD between the YP and XP modes, which is labeled as LD (YP–XP), is large, i.e., >10 dB/cm, over the indicated frequency range. However, the LD between the HO and XP modes, which is labeled as LD (HO–XP), is relatively smaller, especially at the higher frequencies. This behavior occurs because the HO mode for this example case has its E-field distributed mainly throughout the core area and in the air holes. As a consequence, it avoids having a high loss from the lossy ENZ rings. 

### 3.2. Active PCF

Although the ENZ rings introduce a significant LD between the XP and YP modes, the LD between the XP and HO modes is not large enough, as indicated in Figure 3a,b. Moreover, the loss of the wanted mode, i.e., the XP mode, is also rather high, being nearly 10 dB/cm around 1.0 THz. To make this SPSM PCF more practical, the loss of the wanted XP mode must be lowered and the LD values between the XP and HO modes should be increased. These improvements are realized in this work by doping the core area with gain material. The circular gain area of this active PCF is chosen to have a diameter of *d*_3_ = 0.75Λ, where Λ = 70 μm. The doping level is assumed to yield ξ = −0.008. Since the core is only lightly doped with gain material, it barely changes the real part of the refractive index of the core area. Thus, the E-field distributions of the active PCF will remain almost the same as those of the passive PCF shown in Figure 2 and, as a consequence, are not presented here. 

Figure 3c,d plots, respectively, the TLs and LDs of the active PCF example. By comparing Figure 3c with Figure 3a, it is observed that the TLs of all three modes are reduced in the active PCF. This is expected because all the three modes have certain portions of their E-fields distributed over the gain region. By comparing Figure 3d with Figure 3b, it is noted that the LDs (YP–XP) for the passive and active PCFs are similar. This is due to the fact that the portion of the E-field that is distributed in the gain area for the XP and YP modes is similar. On the other hand, the LD (HO–XP) of the active PCF is larger than that of the passive one because the HO mode has its E-field mainly distributed away from the center of the core region, as shown in Figure 2. Consequently, it experiences a smaller amplification than the XP mode does. It is noted that for the active PCF, the TL values can be negative. This means that the amplitude of the corresponding mode is amplified rather than attenuated. To ensure the desired SPSM propagation, the XP mode must then be amplified, but the unwanted modes, i.e., the YP and any HO modes, must be attenuated. 

To facilitate the quantitative assessment of the proposed SPSM PCF, two frequency intervals are defined by the crossover points of the TL curves of the different propagating modes and the TL = 0 line. These intervals are illustrated in Figure 3c. Specifically, the loss of the XP mode is negative (amplified) within interval 1, and the loss of the YP mode is positive (attenuated). Therefore, this interval represents a single-polarization (SP) interval. Similarly, the loss of the XP mode is negative (amplified) in interval 2, and the loss of the HO mode is positive (attenuated). Therefore, this interval represents a single-mode (SM) interval. The overlap of these two intervals is the SPSM’s operational frequency range. In this example, the bandwidths of intervals 1 and 2 are 0.29 and 0.33 THz, respectively, and the overlapping SPSM bandwidth is 0.29 THz. As illustrated by the shaded part of Figure 3d, the MLD value varies from 8.2 to 14.2 dB/cm across this SPSM band. 

## 4. Parameter Study

While a reasonable SPSM bandwidth and MLD value were obtained for the example SPSM PCF, it was found that one can further enhance the performance by tuning its design parameters. The key parameters include the gain factor ξ, the thickness of the ENZ rings *t*, and the diameter of the air holes in first ring *d*_2_. The influence of these parameters on the SPSM PCF’s performance characteristics was studied to optimizethem. 

### 4.1. Gain Factor ξ

The gain parameter ξ is very important to the performance of the active PCF design. It can be adjusted in practice by controlling the amount of gain material doping the core region. A parameter sweep of ξ was conducted, varying its value from −0.006 to −0.01, to reveal its effects on the SPSM bandwidth and MLD values. All other design parameters in this parameter study were fixed as Λ = 70 μm, *d*_1_ = 0.95Λ, *d*_2_ = 0.6Λ, *d*_3_ = 0.75Λ, and *t* = 0.15Λ.

**Figure 4:**
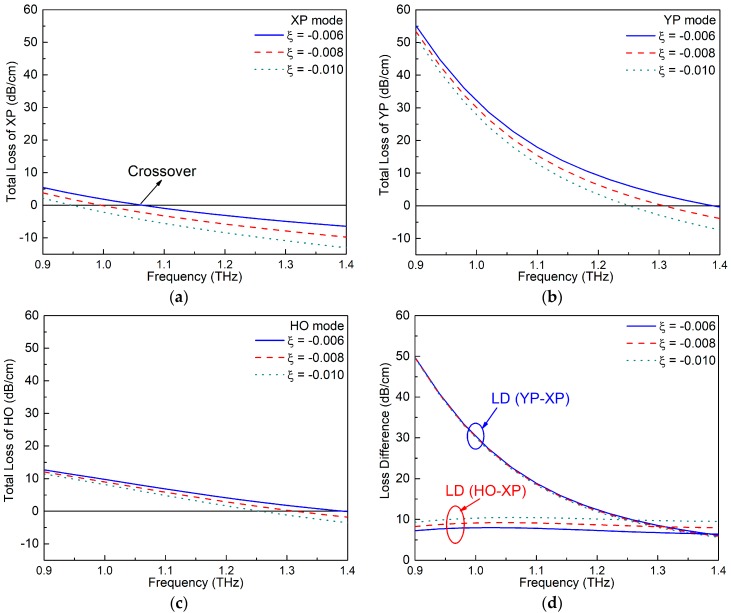
The TL values of the (**a**) XP, (**b**) YP, and (**c**) HO modes of the active ENZ-based PCF as functions of the frequency for different gain factors ξ. (**d**) The corresponding loss difference (LD) values between the wanted and unwanted modes.

Figure 4a–c presents the variations of the TL values of the XP, YP, and HO modes, as functions of the frequency for several ξ values. These results demonstrate that the TL values for all the modes decrease as the absolute value of ξ increases. The variations of the LDs between the wanted and unwanted modes as the frequency was swept are illustrated in Figure 4d. These results show that the LD (YP–XP) remains steady as ξ varies, i.e., the three LD (YP–XP) curves almost completely overlap. This outcome is due to the fact that the two fundamental polarization modes have similar portions of E-field distributions in the circular gain area. Therefore, the amplification rates for these two modes are similar no matter what the value of ξ is. On the other hand, as Figure 2 illustrates, the portion of the E-field distribution of the HO mode is smaller in the gain area when compared to the XP mode. This means the XP mode will have a greater benefit from the presence of gain in the core area and explains why the LD (HO–XP) increases as ξ increases, as shown in Figure 4d. 

Furthermore, changing ξ leads to different crossover points and, hence, different SPSM bandwidths. The specific values of the crossover points, interval range, SPSM bandwidth, and MLD values for each ξ value are compiled in Table 1. The up and down arrows are used to represent the tendency of the listed parameters to vary as ξ increases. The tables clearly show that the two intervals and the SPSM bandwidth decrease while the MLD value increases as ξ increases. With an overall consideration of the bandwidth and the MLD value, ξ = −0.008 was selected as the optimal value. 

### 4.2. ENZ Ring Thickness t

**Figure 5:**
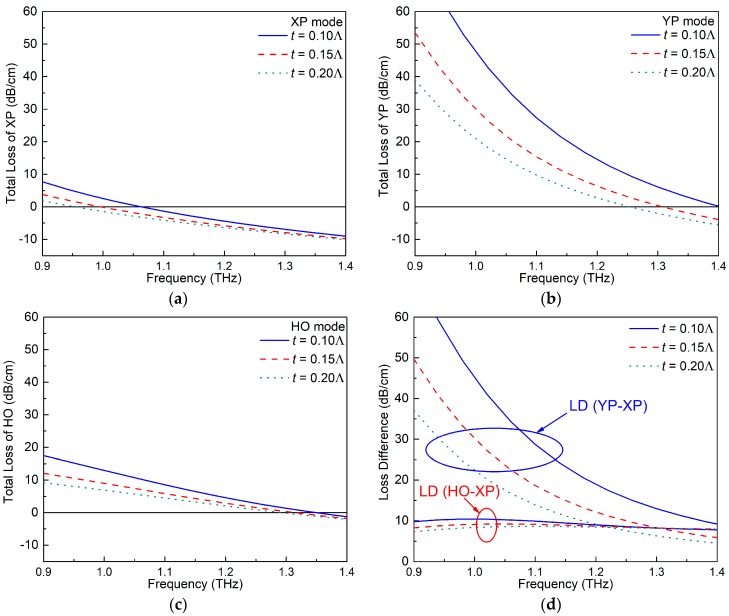
The TL values of the (**a**) XP, (**b**) YP, and (**c**) HO modes of the active ENZ-based PCF as functions of the frequency for different ENZ ring thicknesses (*t*). (**d**) The corresponding LD values between the wanted and unwanted modes.

Another important parameter is the thickness of the ENZ rings *t*. This thickness strongly impacts the portion of the E-field distribution that lies inside the ENZ rings and, hence, the LD values for the various modes. A parameter sweep of *t* was conducted from 0.1Λ to 0.2Λ, with the other parameters fixed as Λ = 70 μm, *d*_1_ = 0.95Λ, *d*_2_ = 0.6Λ, *d*_3_ = 0.75Λ, and ξ = −0.008. Figure 5a–c shows the variations of the TL values of the XP, YP, and HO modes, with frequencies for different values of *t*. The corresponding variations of the LD values are presented in Figure 5d. 

**Figure 6:**
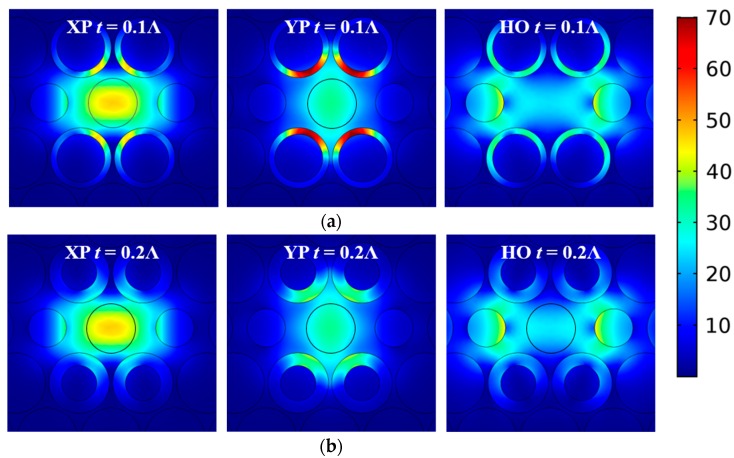
Simulated distributions of the magnitude of the total electric field of the XP, YP, and HO modes at 1.0 THz of the active ENZ-based PCF, with a ring thickness of (**a**) *t* = 0.1Λ and (**b**) *t* = 0.2Λ. (The scale bar is the same for all subplots.).

Figure 5a–c clearly demonstrates that the TL values for all of the modes are larger when the ENZ rings are thinner. To understand this behavior, the magnitude of the total E-field distributions of the three modes when *t* = 0.1Λ and 0.2Λ are shown in Figure 6a,b, respectively. As illustrated, the E-field in the ENZ rings is larger in the smaller thickness *t* case for all three mode types. From the electromagnetic interface conditions for the electric field component normal to the interface, $\varepsilon_{ENZ}E_{n,ENZ}=\varepsilon_{outside}E_{n,outside}$, and the components tangential to the interface, $E_{t,ENZ}=E_{t,outside}$, it is clear that the magnitude of the total electric field will always be larger in the ENZ region for all modes. Thus, the majority of the attenuation is associated with lossy ENZ rings since the loss is proportional to $\iint \sigma \;E_{total}^{2}\;ds$, and the combined EML in the air and HRS regions is tiny. The thinner ENZ rings have even larger total electric fields because the electric fields near the two interfaces are essentially in-phase (the index of refraction of an ENZ region is nearly zero) and are nearly of the same magnitude. 

Since the ENZ-augmented air holes were taken to be along the y-axis, the thickness of the ENZ rings has the strongest effect on the TL value of the YP mode. On the other hand, the XP and HO modes have much smaller E-fields distributed in the ENZ rings. Therefore, the TL values of the XP and HO modes are much smaller than those of the YP mode, and changes in *t* have very similar small effects on them. This behavior is in accordance with the fact that the LD (YP–XP) values change significantly in Figure 5d, but the LD (HO–XP) values remains quite stable for different *t* values. 

The specific values of the crossover points of each mode, the interval size, the SPSM bandwidth, and the MLDs for different values of *t* are listed in Table 2. Since the case *t* = 0.15Λ provides the widest SPSM bandwidth and the highest MLD value, it was selected to be the optimal choice.

### 4.3. The Diameter d_2_ of the Air Holes Alongside the Gain Region

**Figure 7:**
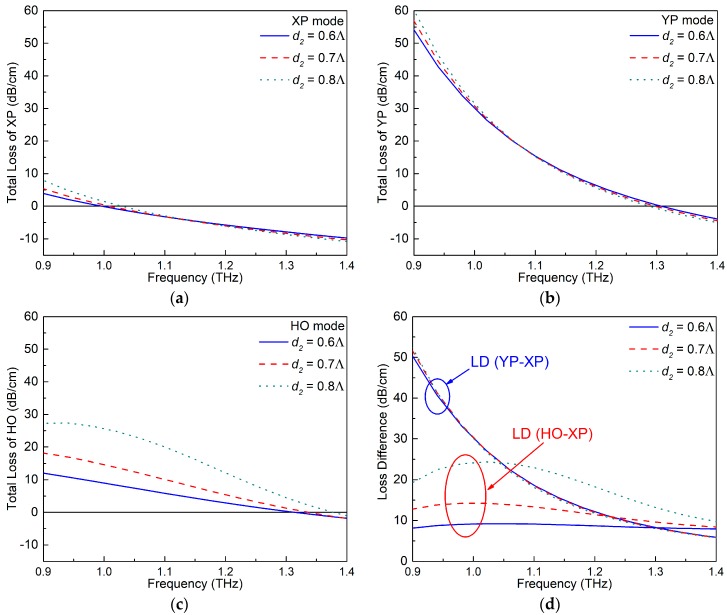
The TL values of the (**a**) XP, (**b**) YP, and (**c**) HO modes of the active ENZ-based PCF as functions of the frequency for different diameter *d*_2_. (**d**) The corresponding LD values between the wanted and unwanted modes.

**Figure 8:**
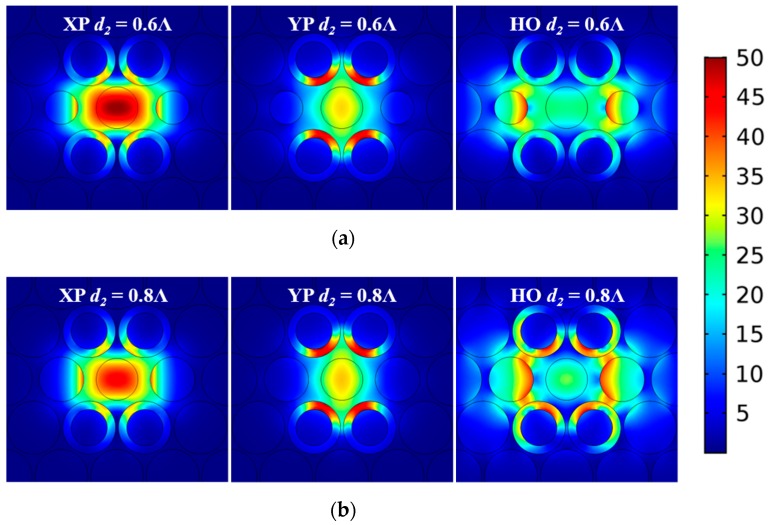
Simulated distributions of the magnitude of the total electric field of the XP, YP, and HO modes of the active ENZ-based PCF at 1.0 THz when (**a**) *d*_2_ = 0.6Λ and (**b**) *d*_2_ = 0.8Λ. (The scale bar is the same for all subplots.)

According to Figure 2, the E-field distribution of the HO mode is concentrated near the air holes alongside the gain area. Therefore, it was expected that changing the diameter of the air holes, *d*_2_, would also affect the E-field distribution of the HO mode in the active case. A parameter sweep of *d*_2_ was conducted from 0.6Λ to 0.8Λ while keeping the other parameters fixed as Λ = 70 μm, *d*_1_ = 0.95Λ, *d*_3_ = 0.75Λ, ξ = −0.008, and *t* = 0.15Λ. Figure 7a–c shows the TL values of the XP, YP, and HO modes as functions of the frequency for different values of *d*_2_. The corresponding variations of the LD values between the wanted and unwanted modes are given in Figure 7d. The E-field distributions of these three modes for *d*_2_ = 0.6Λ and 0.8Λ are illustrated in Figure 8. 

As shown in Figure 7a–c, the variation of *d*_2_ leads to noticeable changes to the TL values of the HO mode, but it has only minimal effects on those of the XP and YP modes. This is confirmed by the E-field distributions shown in Figure 8. As the diameter *d*_2_ of the air holes becomes larger, the portion of the E-field distribution of the HO mode remains essentially the same in the gaps between the air holes but is squeezed into the ENZ rings, resulting in higher TL values. On the other hand, the confinement ability of the PCF increases as the frequency increases. This mitigates the effect introduced by changing *d*_2_ at those higher frequencies. These behaviors explain the variations of the LD values depicted in Figure 7d. 

The specific values of the crossover points of each mode, the interval sizes, the SPSM bandwidth, and the MLD values for different values of *d*_2_ are summarized in Table 3. Note that although a larger *d*_2_ leads to better MLD performance, the bandwidth becomes narrow. Consequently, the diameter *d*_2_ = 0.6Λ was chosen as the optimal value. This value allows the PCF to obtain a wider SPSM bandwidth while maintaining similar MLD values. It is noted that a larger MLD value in the lower frequency range can be obtained if needed by choosing larger *d*_2_ values (as illustrated in Figure 7d) and sacrificing some bandwidth. 

### 4.4. Optimized Results

Upon completion of the parameter studies, the optimized parameters of the PCF were obtained. They parameters are Λ = 70 μm, *d*_1_ = 0.95Λ, *d*_2_ = 0.6Λ, *d*_3_ = 0.75Λ, ξ = −0.008, and *t* = 0.15Λ. With these optimal dimensions, the active SPSM PCF has a wide SPSM bandwidth of 0.312 THz from 0.996 to 1.308 THz and a large MLD value (greater than 8.0 dB/cm) across the entire SPSM band. Compared to the SPSM bandwidth of the PCFs reported in [13] and [14] (respectively 0.13 and 0.32 THz), the SPSM bandwidth attained here is wider and comparable, respectively. Nevertheless, the MLD values of the PCF reported in [13] and [14] were only 0.036 dB/cm and 0.06 dB/cm, respectively, at 1.0 THz. In comparison, a remarkable improvement on the MLD value, >8.0 dB/cm, was achieved here.

### 4.5. Fabrication Issues

With the development of hybrid optical fiber techniques [19], various complex materials, such as gases, liquids, metals, semiconductors, and even metamaterials, can now be integrated into a PCF. Taking advantage of these techniques, direct thermal drawing, pressure-assisted melt filling, and high-pressure chemical vapour deposition [19,29,30,31] could facilitate the insertion of naturally occurring bulk ENZ materials into a structured fiber. Since the optimized design would need the deposition of four ENZ rings in the selected air holes, the most probable implementation technique to realize it would be high-pressure chemical vapour deposition [19,31]. The potential ENZ material choices that are currently available would be either the high purity bulk form of KCL [17] or a specifically tailored metamaterial [32]. We also are currently exploring the possibility of using additive manufacturing techniques to realize a prototype for testing purposes.

As expected with the fabrication of any PCF fiber, the exact design parameters will be imperfectly realized. These imperfections could have a dramatic effect on the predicted performance characteristics. However, the simulations associated with the described parameter studies indicate that the optimized design is very tolerant to these imperfections. According to Table 1, Table 2 and Table 3, the performance variations would still be acceptable, even with significant changes to those key dimensions. In fact, the bandwidth would be reduced, at most, from 0.312 to 0.259 THz, and the MLD from 8.0 to 6.4 dB/cm, in the worst cases. These performance characteristics are still acceptable and remain much better than the ones associated with the previously reported THz SPSM PCFs. Since any actual changes in the design dimensions from fabrication errors would be much smaller than those worst cases, only minor performance degradation is expected from any fabrication related imperfections.

**Figure 9:**
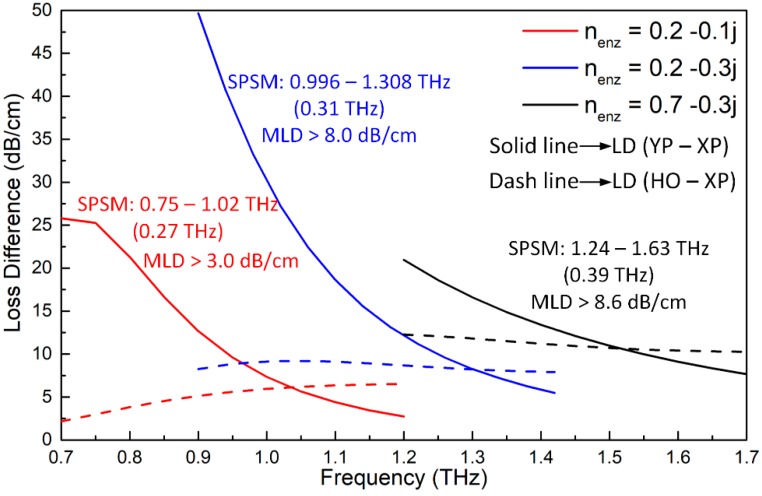
The LD values of the active ENZ-based PCF as functions of the frequency for different refractive index values, *n_enz_*.

The influence of the potential variations of the refractive index of the realized ENZ material with a frequency (e.g., dispersion) was also investigated. As shown in Figure 9, even with significant changes in the refractive index, the performance characteristics associated with the optimized PCF remain acceptable. For the worst case, where *n_enz_* = 0.2–0.1 j, the SPSM bandwidth, 0.27 THz, is, again, reasonable. Furthermore, the MLD remains above 3.0 dB/cm, which, again, is much better than the values of the previously reported THz SPSM PCFs. Even though we have not found any specific materials that have ENZ properties over the lower portion of the THz frequency band, it is anticipated that many more ENZ materials will become available in the near future due to the strong recent interest in them [33,34]. These materials will make the realization of the reported THz SPSM PCF possible.

## 5. Conclusions

This paper demonstrated a novel PCF design that achieves a very wide SPSM bandwidth and a large loss difference between the wanted (XP) and unwanted (YP and HO) modes. While the geometrical configuration is quite simple, the significantly improved MLD values between the wanted and unwanted modes was achieved by manipulating their EMLs. This behavior was realized by introducing ENZ rings into selected air holes of the cladding and doping the core area with gain material. The working mechanism was illustrated by the passive and active PCF examples. Parameter sweeps of the key design parameters were discussed to demonstrate how the optimized SPSM PCF was obtained. The optimized active SPSM PCF had a wide SPSM bandwidth of 0.312 THz from 0.996 to 1.308 THz and an MLD value greater than 8.0 dB/cm over that frequency range. The reported PCF can be used to maintain a single propagating fundamental polarization mode while suppressing all other modes. By using only a short segment with a length of 2.5 cm or 5.0 cm, the MLD values between the wanted mode and the other unwanted modes of the reported SPSM PCF exceed 20 dB or 40 dB, respectively.

## Figures and Tables

**Table 1:** Crossover points, interval size, SPSM bandwidth, and minimum loss difference (MLD) values for different gain factors, ξ.

Gain Factor	Crossover X (THz)	Crossover Y (THz)	Crossover HO (THz)	Interval 1 (THz)	Interval 2 (THz)	SPSM Bandwidth	MLD (dB/cm)
−0.006	1.065	1.388	1.394	0.323	0.329	0.323	>6.4
−0.008	0.996	1.308	1.315	0.313	0.32	0.312	>8.0
−0.01	0.948	1.252	1.257	0.304	0.309	0.304	>9.8

The arrows represent the tendency to increase or decrease as ξ increases.

**Table 2:** Crossover points, interval size, SPSM bandwidth, and MLD values for different ENZ ring thicknesses (*t*).

*t*	Crossover X (THz)	Crossover Y (THz)	Crossover HO (THz)	Interval 1 (THz)	Interval 2 (THz)	SPSM Bandwidth	MLD (dB/cm)
0.1	1.062	1.404	1.349	0.342	0.287	0.287	>8.1
0.15	0.996	1.308	1.315	0.313	0.319	0.312	>8.0
0.2	0.954	1.253	1.292	0.299	0.338	0.299	>7.5

The arrows represent the tendency to increase or decrease as *t* increases.

**Table 3:** Crossover points, interval size, SPSM bandwidth, and MLD values for different air hole diameters (*d*_2_).

*d*_2_	Crossover X (THz)	Crossover Y (THz)	Crossover HO (THz)	Interval 1 (THz)	Interval 2 (THz)	SPSM Bandwidth	MLD (dB/cm)
0.6 Λ	0.996	1.308	1.315	0.313	0.319	0.312	>8.0
0.7 Λ	1.01	1.297	1.337	0.287	0.327	0.287	>8.2
0.8 Λ	1.029	1.288	1.378	0.259	0.349	0.259	>8.3
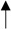	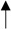	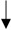	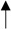	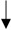	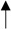	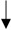	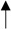

The arrows represent the tendency to increase or decrease as *d*_2_ increases.
